# Teaching an old dog new tricks: reactivated developmental signaling pathways regulate ABCB1 and chemoresistance in cancer

**DOI:** 10.20517/cdr.2020.114

**Published:** 2021-06-19

**Authors:** Wing-Kee Lee, Thévenod Frank

**Affiliations:** Institute for Physiology, Pathophysiology and Toxicology, ZBAF, Witten/Herdecke University, Witten 58453, Germany.

**Keywords:** Drug resistance, ABC transporters, transforming growth factor beta, tumor heterogeneity, tumor cell biology

## Abstract

Oncogenic multidrug resistance (MDR) is a multifactorial phenotype intimately linked to deregulated expression of detoxification transporters. Drug efflux transporters, particularly the MDR P-glycoprotein ABCB1, represent a central mechanism by which not only chemotherapeutic drugs are extruded or sequestered to prevent drug delivery to their intracellular targets, but also for inhibiting apoptotic cell death cues, such as removal of proapoptotic signals. Several cell populations exhibiting the MDR phenotype co-exist within a tumor, such as cells forming the bulk tumor cell mass, cancer stem cells, and cancer persister cells. The key to regulation of ABCB1 expression is the cellular transcriptional machinery. Developmental signaling pathways (e.g, Hedgehog, Notch, Wnt/β-catenin, TGFβ, PITX2) are pivotal in governing cell proliferation, survival, differentiation and guiding cell migration during embryogenesis, and their reactivation during carcinogenesis, which is of particular significance for tumor initiation, progression, and metastasis, also leads to the upregulation of ABCB1. These pathways also drive and maintain cancer cell stemness, for which ABCB1 is used as a marker. In this review, the contribution of canonical and non-canonical developmental signaling pathways in transcriptional regulation of ABCB1 to confer MDR in cancer is delineated.

## Introduction

Bacterial resistance to antibiotic drugs was first described after the discovery that penicillin prompted bacteria to develop several important defense mechanisms, including the expression of efflux transporters in the outer cell wall. The broad range of substrates used by these transport proteins resulted in coining the term multidrug resistance (MDR) as pathogens can limit the accumulation of drugs targeted against them^[1]^. Widespread use of chemotherapeutic drugs in cancer therapy resulted in the evolution of an analogous program in mammalians, and the often-reported human form of MDR in tumor cells. According to patient records, the first human case of resistance to chemotherapy was observed in 1942 during the use of nitrogen mustard gas to treat a patient with recurrent lymphosarcoma^[2]^.

The discovery of a molecular link strongly associated with the MDR phenotype: P-glycoprotein (“altered drug permeability glycoprotein”), abbreviated as P-gp, gp170, P170 or, according to current nomenclature, ABCB1 (ATP-binding cassette sub-family B member 1) was achieved by exposure of non-drug resistant cell lines to increasing concentrations of cytotoxins^[3]^. In colchicine-resistant Chinese hamster ovary cells, a membrane glycoprotein absent in parental cells was associated with an ostensible “permeability barrier” to colchicine toxicity^[4]^. This glycoprotein was further characterized and designated as P-gp based on its apparent ability to control drug permeation by modulating properties of hydrophobic membrane regions in drug-resistant cells^[5]^. In the first instance, the druggability of ABCB1 was promising because it represented a molecular link to MDR. Drug development has spawned three generations of ABCB1 inhibitors, which are usually effective in reversing chemoresistance in cell lines but are hampered in *in vivo* models or human clinical trials by physiological *ABCB1* expression at barrier and excretion sites, culminating in adverse side effects. Development of the “fourth generation” ABCB1 modulators is focused on already available natural compounds^[6]^, yet it is becoming increasingly apparent that MDR is a multifactorial phenomenon that will require targeting of a mechanism underlying several MDR-contributing factors^[7,8]^.

ABCB1-dependent MDR commonly involves regulation of expression, such as gene transcription, epigenetics, or post-translational modifications^[7,9]^. Due to ABCB1’s indispensable roles in tissue protection and detoxification, its expression follows developmental patterns in the fetus as the key sites for ABCB1 begin to develop. These include endothelium of the blood-brain-barrier, proximal tubule of the kidney, intestinal epithelium, and liver hepatocytes. Little is known about ABCB1 expression during gestation; it is presumably under the control of transcription factors belonging to developmental signaling pathways that orchestrate the intricate and highly choreographed process of cell growth and patterning^[10,11]^.

With exception of some specialized tissues, developmental signaling pathways such as Hedgehog, Notch, and Wnt, are switched off and remain dormant in adult life. However, they can be aberrantly reactivated during carcinogenesis^[12-14]^, wherein cellular proliferation and migration becomes uncontrolled. Upregulation of ABCB1 through these signaling pathways poses a major hurdle because not only is it pivotal to tumor MDR, but it is also a major contributor to the survival of self-renewing tumor cells that are capable of regenerating a tumor after treatment, and thus a marker of cancer cell stemness.

In this review, the resurgence of signaling activity of pathways classically associated with development of oncogenic ABCB1-dependent MDR will be discussed. Also, the discovery of the HOX gene and homeodomain protein PITX2 (Paired Like Homeodomain 2) as a positive ABCB1 regulator will be presented.

## ABCB1

Physiological expression of ATP-binding cassette (ABC) transporters, such as multidrug resistance (MDR) P-glycoprotein MDR1/*ABCB1*, multidrug resistance related protein MRP1/*ABCC1*, and breast cancer resistance protein 2 BRCP2/ABCG2, at barrier sites, including colon, liver, kidney, and blood-brain barrier, serve to protect against xenobiotics and toxic metabolites^[15]^. The importance of ABCB1 in protection from damaging agents is highlighted by an anecdote associated with the generation of the *Abcb1a*^-/-^ mouse. Though the mouse was viable and showed no functional deficiency, it was far more sensitive to chemical challenges as exemplified by an accidental observation where the centrally-neurotoxic pesticide, ivermectin, proved lethal to *Abcb1a*^-/-^, but not *Abcb1*^+/+^, mice due to compromised blood-brain barrier function^[16]^.

The canonical MDR protein, ABCB1, belongs to the evolutionary-conserved ABC superfamily, a large group of transmembrane (TM) transporters that utilize energy to translocate various substrates across membranes. At the plasma membrane, ABCB1 extrudes a broad range of structurally unrelated substrates directed towards the extracellular space. Substrate suitability for ABCB1 is determined and limited by size, charge, and hydrophobicity; neutral or cationic compounds ranging from 300-4000 Da and compounds with high hydrophobicity are favored, though amphiphilic compounds are also translocated^[17-20]^. Moreover, ABCB1 is a lipid translocase with broad specificity^[21]^, extruding simple phospholipids and sphingolipids^[22,23]^.

### Structure and function of ABCB1

#### ABCB1 structure

Full length human ABCB1 was cloned in 1986^[24,25]^, but it required more than a decade to visualize the tertiary structure of mouse ABCB1 by X-ray crystallography^[26]^. Functional ABC transporters typically consist of at least two TM domains (TMDs) and two nucleotide binding domains (NBDs)^[17]^. In humans, ABCB1 protein is synthesized from the *ABCB1* gene (previously *MDR1*) generating a single polypeptide chain that is folded to form two homologous parts, each consisting of six a-helical TMDs and an NBD, joined by a cytoplasmic linker region. The α-helical TMDs of each homologous half create a bundle that comes together to form a large internal cavity, which can easily accommodate two compounds^[27]^. In the resting state, ABCB1 is found in a triangular conformation where the extracellular bundle “heads” are close to each other. The substrate-binding pocket faces inward to accept compounds only from intracellular origin, that is, the cytoplasm and inner membrane leaflet (IML) of the lipid bilayer, but not from the extracellular compartment or outer membrane leaflet (OML)^[28]^. The predicted substrate-binding pocket contains mostly hydrophobic and aromatic residues, and each substrate has a defined amino acid subset for its recognition^[29]^.

#### ABCB1 transport function

In the catalytic cycle, substrate binding stimulates ATP binding to the NBDs, leading to their dimerization, which in turn, drives the large structural change of ABCB1 from an inward to outward-facing conformation. The substrate is then released on the opposite side of the membrane through decreased binding affinity, facilitated by ATP hydrolysis or by the law of mass action. Following hydrolysis of ATP, NBD dimerization is disrupted and ABCB1 returns to the pre-transport state^[26]^.

Structural and mutational studies reveal potential mechanisms by which ABCB1 may extrude a wide variety of structurally unrelated substrates^[17]^. The “vacuum cleaner” model describes recognition, extraction, and extrusion of hydrophobic cationic substrates partitioned in the hydrophobic portion of the IML^[28,30,31]^. However, this model is increasingly challenged because ABCB1 transport kinetics do not follow Michaelis-Menten kinetics. An alternative “oscillation” hypothesis has been proposed: continuous interchange between inward and outward conformations results in the stochastic extrusion of substrates translocated to the IML by flippase activity^[32]^ or passive distribution via lateral diffusion^[33]^. According to this model, effective ABCB1 inhibitors likely lock ABCB1 in the outward conformation and thereby hinder substrate extrusion.

Garnering evidence supports both the vacuum cleaner and oscillation models^[34,35]^. The partition coefficient of a substrate into the lipid bilayer, dependent on its chemistry, is an important determinant of ABCB1 affinity and thus transport^[36]^. Interestingly, in addition to the hydrophobic core of the membrane, ABCB1 potentially binds substrates that partition at the cytoplasmic surface of the membrane^[34,37,38]^. For extrusion, the molecule laterally diffuses to the substrate-binding chamber of ABCB1 driven by its hydrophobic residues, and without direct involvement of the hydrophobic core. Taken together, efflux of substrates by ABCB1 is a multistep process involving (1) initial substrate binding to membrane lipids; (2) entry; partitioning into the lipid bilayer/interfacial region or flippase-driven movement into the IML (3) recognition by ABCB1; (4) uptake from the hydrophobic bilayer core or interfacial region, and binding pocket entry; and finally (5) transport to the opposite/extracellular compartment through a conformational change of ABCB1 facilitated by ATP hydrolysis. Thus, an apparent combination of lipid binding and ABCB1 substrate recognition forms the basis of ABCB1 efflux function.

### ABCB1 upregulation in chemoresistant cancer cells

Ineffective chemotherapy treatment is one of the greatest challenges posed by modern cancer treatment, which in part, results from upregulation of broad range transporters such as ABCB1, in many cancer cells. High expression of ABCB1 in tumors is associated with increased cell survival due to efficient extrusion of chemotherapeutic drugs, evasion of apoptosis, and increased metastatic potential, resulting in poor prognosis^[9,39]^. As detailed below, levels of ABCB1 are predominantly regulated by transcription factors^[40]^ such as developmental transcription factors TCF4 (T-cell factor 4)^[41]^ or PITX2^[42,43]^ that have become oncogenic.

#### Intracellular localization of ABCB1

Drug resistance is not only determined by the expression of ABCB1 in the plasma membrane, wherein ATP-dependent drug efflux to the extracellular space prevents its accumulation in the cell, but also by the intracellular expression of functional ABCB1^[44-46]^ that further restricts drugs from entering the nucleus where drug targets are commonly localized. Cytoplasmic ABCB1 expression was identified in drug resistant gastric carcinoma cells with immunohistocytochemistry^[47]^. Using pulse chase experiments and tracking of autofluorescent daunorubicin, an anthracycline chemotherapeutic drug, nucleus-derived fluorescent vesicles appeared to be trafficked to the cell periphery followed by their exocytosis^[47]^. Similar findings were reported in LLC-PK1 renal proximal tubule cells^[48]^, and the acidic nature of the ABCB1 expressing or drug-accumulating vesicles was revealed^[45,48,49]^. The presence of functional ABCB1 in vesicular structures was confirmed using immunostaining, which ruled out endocytic vesicles and fluorescent substrate accumulation^[44]^. The origin of ABCB1/drug-accumulating vesicles is still unclear. An acidic vesicular pool that accumulates anthracycline drugs is well-defined and accumulating evidence points towards lysosomes as a potential target, wherein ABCB1 is localized as part of its degradation/turnover cycle^[50]^ and internalized into after tumor micro-environment stress signals, such as hypoxia or nutrient starvation^[51]^. Moreover, lysosome biogenesis was found to be induced, facilitating mislocalization of doxorubicin to lysosomes and consequent drug resistance^[51,52]^.

A further line of defense against nuclear drug accumulation has been evidenced directly in the nuclear envelope^[46,53,54]^. A different glycoside chain could explain the targeted trafficking of ABCB1 to the nucleus^[54]^. Kinetic studies have suggested that vesicles bud from the nuclear envelope to expel drugs from the cell through exocytosis^[47]^, whereas direct nuclear-to-cytosol efflux activity of ABCB1 might be affected by local ATP concentrations and lipid composition of nuclear membranes.

#### Lipid microenvironment in ABCB1 functionalization

Integral membrane proteins often exhibit lipid binding specificity and are dependent on their lipid microenvironment for correct function. Functionalization of ABCB1 is determined not only by the composition of lipids in its immediate vicinity but also by fluidity, phospholipid headgroup, and fatty acyl chain length (reviewed in^[55]^). Small, specialized membrane domains (< 100 nm), also known as lipid rafts (LRs), are highly enriched in cholesterol, glycosphingolipids, and phospholipids with saturated fatty acids, particularly SM, and provide an optimal lipid microenvironment for integral membrane protein function (reviewed in^[56,57]^). ABCB1 localizes to LRs^[58,59]^, possibly due to its capacity to bind cholesterol^[60]^ or SM^[8]^. Cholesterol depletion reduces ABCB1 transport activity, which is associated with migration of ABCB1 out of LRs, indicating ABCB1 activity dependence on LR localization^[61,62]^. In support, cholesterol repletion enhances ABCB1 activity^[62]^ and movement of substrates across a lipid membrane is more efficient when cholesterol and SM are present^[63]^.

### Transcriptional regulation of ABCB1

ABCB1 expression is predominantly ruled at the transcriptional level. The eukaryotic transcriptional machinery is a large multi-protein complex, comprised of (co-)activators, (co-)repressors, polymerases, and more^[64,65]^. DNA accessibility and responsiveness are additional crucial factors for the transcriptional machinery to bind to target DNA sequences and initiate or repress gene expression.

The promoter region of ABCB1 contains multiple sites for activating transcription factors, including myc, Sp1, AP-1, nuclear factor kappa B (NF-κB), TCF (reviewed in^[40]^), and the more recently-discovered, Sp3^[66]^, octamer-binding transcription factor 4 (OCT-4)/POU5F1^[67]^ and PITX2^[42,43]^. Repression of ABCB1 expression can occur through the binding of oncogenic chimeric proteins^[68,69]^, DNA methylation^[70,71]^ or a combination of transcription factors, for example, NF-κB with c-Fos^[72]^. Furthermore, recent findings in drug resistant ovarian and breast cancers showed evidence of multiple aberrant transcriptional fusions of ABCB1, placing it under control of alternative promoter regions from genes in chromosomal proximity to ABCB1^[73]^. These data point towards non-canonical mechanisms of transcriptional regulation that broaden the range of ABCB1 regulators and thus increasing disposition to MDR progression. The full impact of upstream signaling pathways in regulating ABCB1 transcriptional fusions is yet to be clarified. Thus, elevated ABCB1 levels can be controlled through several ways, including increased activator activity, decreased repressor activity, chromatin remodeling^[74]^, DNA priming^[75]^, and transcriptional fusions^[76]^.

Since many of the aforementioned transcription factors are altered by stressors, ABCB1 is highly susceptible to upregulation during a stress response. ABCB1 expression can also be modified by single nucleotide polymorphisms (SNPs)^[77,78]^, which play an important role in cancer therapy, gene rearrangements^[79]^, mutations^[17,80]^, epigenetic regulation^[81,82]^, and post-translational mechanisms^[83]^.

### ABCB1 expression in chemoresistant cancer stem cells

#### Cancer stem cells

Intratumor heterogeneity stems from various intratumor cell populations^[84]^ and/or spatial heterogeneity between intratumor structures and the tumor microenvironment^[85]^. Tumor initiating or cancer stem (-like) cells (CSCs) possess self-renewing capacity, ability to differentiate into multiple tumor cell types, and display enhanced resistance to chemotherapy and apoptosis cues^[86-89]^. In solid tumors, they were first identified, isolated, and characterized by presence of the stem cell marker, CD133, in the brain^[90]^. During therapy, some tumor cell populations are incapable of defending themselves and undergo cell death, whereas CSCs are well equipped with defense mechanisms such as altered drug transporter expression, which render them impervious to the death cues proffered by anticancer drugs^[91]^. Due to the small number of CSCs in a tumor, they are often below detectable levels and may remain dormant for a significant time (i.e, years to decades). The existence of CSCs has been much debated^[92]^ and could be limited to certain hemopoietic cancers and some solid tumors. Several hypotheses exist for the origin of CSCs, such as cell fusion, de-differentiation of tumor cells, or genomic instability (reviewed in^[93]^).

Since their discovery, a multitude of CSC markers have emerged. Aside from CD133^+^, which is also found in persister cancer cells^[94]^, CD24^-^, CD44^+^, ABCB1^+^, ABCG2^+^ and ALDH^+^ are common phenotypic markers for CSCs. Because of the challenges in identifying expression patterns of cell surface CSC-specific markers across all CSCs from different cancer tissues, current methodologies take advantage of the ABC transporter substrate Hoechst-33342 (Hoechst) combined with flow cytometry analysis to isolate the so-called side population. Since CSCs express high ABC transporter levels, there is less accumulation of Hoechst dye in the cells, which can be detected through cellular fluorescence intensity^[95]^.

#### ABCB1 in cancer stem cells

The challenge of eradicating MDR cancers lies not only in tumor cells that do not respond to chemotherapeutic therapy and continue to proliferate, but also encompasses the tenacity of CSCs^[96]^ that are drug-tolerant and contribute to tumor repopulation. CSCs undergo genetic changes such as increased expression of antiapoptotic Bcl-2 and multiple ABC drug transporters, causing acquisition of apoptosis resistance and chemoresistance, respectively^[89,97,98]^.

ABCB1^+^ CSCs have been isolated from non-small cell lung cancer^[99]^, ovarian cancer^[100-102]^, colorectal cancer^[103-105]^, pancreatic cancer^[106]^, oral squamous cell carcinoma^[107]^ and glioblastoma^[108,109]^. Similar to tumor cells and epithelial cells, ABCB1 expression (mRNA and protein) in CSCs is largely governed by transcriptional regulation, which has been demonstrated for Kelch-like ECH-associated protein 1 (KEAP) with nuclear factor erythroid 2-related factor 2 (NRF2)^[110]^, OCT-4/POU5F1^[67,111]^, NANOG/STAT3^[112]^, Twist^[113]^, and Wnt/β-catenin^[114]^. Intriguingly, expression or activation of surface markers CD133^+^^[115]^ or CD44^+^^[112]^, respectively, engages ABCB1-elevating signal transduction, indicating that stemness is inherently linked to chemoresistance.

## Developmental signaling pathways

A complicated, highly choreographed series of molecular events orchestrate cell lineage commitment, cell expansion, cell differentiation, organ asymmetry, positioning, patterning, and organism sculpturing during embryonic development^[116-118]^. Developmental signaling pathways usually remain dormant in adult life but are frequently reactivated during carcinogenesis, contributing to cell survival, proliferation, resistance to apoptosis and MDR^[12-14]^. With a limited combination of receptors and ligands, the pleiotropic effects of gene transcription targeted by developmental signaling need to be adequately communicated. Aside from correct timings, receptor-ligand interactions are finely tuned and highly discriminating, such that even slight variations in the associated interaction dynamics and kinetics have a profound effect on signal transduction. These communication codes encompass differences in signal amplitude, frequency, duration, fold change, ligand combinations, ligand concentrations, and kinetics of ligand binding (reviewed in^[119]^).

## Classical developmental signaling pathways in ABCB1 regulation in cancer

### Notch signaling

In contrast to other developmental signaling pathways, which are engaged following docking of a secreted ligand to cell surface receptors, the Notch pathway is activated through ligand-receptor binding of contiguous cells. Notch signaling is active in several developmental programs, mostly in the ones that determine cell differentiation, but also in the cell proliferation and stem cell maintenance programs in tissues such as the heart, central nervous system, and pancreas (reviewed in^[120]^).

#### Canonical Notch signaling

Four receptors (Notch1-4) and multiple ligands (Delta, Jagged) have been identified in mammalians. Notch receptors are type I membrane proteins with a single transmembrane pass. Upon docking of the ligand onto the Notch receptor, a conformational change permits a successive two-step cleavage event in a process termed regulated intramembrane proteolysis^[121-124]^. The first and rate-limiting step involves matrix metalloproteases (ADAMs, A Disintegrin And Metalloproteinases), which dissociate the extracellular and intracellular domains of Notch receptor at the S2 cleavage site. The Notch extracellular domain (NECD) is shed from the membrane and the cleavage product Notch Extracellular Truncation (NEXT) remains membrane bound. The second step requires scission in the transmembrane domain by the γ-secretase complex^[125,126]^ at the S3/4 cleavage sites, releasing the Notch intracellular domain (NICD) from the membrane into the cytoplasm and permitting its translocation to the nucleus. Endocytosis of the Notch receptor occurs and is crucial for signal transmission. Moreover, Notch signals can be conveyed differentially through the generation of different products that have varying signal duration and downstream targets^[127]^, allowing for the communication of distinct ligand-receptor combinations^[119]^.

Another level of regulation exists in the cytoplasm. There, NICD turnover is controlled by modification of the C-terminal PEST domain, which is rich in proline (P), glutamic acid (E), serine (S), and threonine (T). Generally, phosphorylation and ubiquitination affect degradation, whereas hydroxylation and acetylation affect the half-life of NCID^[127]^. Once NICD reaches the nucleus, it binds to CSL/RBPjK (CBF-1, Suppressor of Hairless, Lag-2/Recombination signal Binding Protein for immunoglobulin Kappa J region) and regulates gene transcription. Pertinent to Notch’s multifaceted role in embryonic development, not one set of genes can be assigned to Notch that is regulated in the same way in every cell or by activation mode, adding to Notch’s complexity and diversity. Common gene signatures include the HESR (Hairy and Enhancer of Split-Related) genes, which encode basic helix-loop-helix-type transcriptional repressors, c-myc, cyclin D1, and Snail^[127]^.

#### Non-canonical Notch signaling

Notch signaling independent of CSL in both ligand-dependent and independent manners are considered to be non-canonical and is often associated with disease^[128]^. Increase in NICD (cytoplasmic or membrane tethered) is usually prerequisite and rather than directly altering gene transcription, NICD interacts with components of other signaling pathways such as Akt/PKB (protein kinase B), Yin-Yang-1 (YY1), NF-κB, β-catenin or hypoxia-inducible factor 1-α (HIF-1α), to control transcription (reviewed in^[128]^). The interaction between Notch and β-catenin has been best described - both NCID and uncleaved Notch receptor can directly interact with the active form of β-catenin, repressing its activity, either by sequestration or by targeting β-catenin to the endo-/lysosomal compartment by endocytosis, respectively (reviewed in^[129]^).

#### Regulation of ABCB1 by Notch

Notch signaling has been positively linked to ABCB1 expression in ovarian cancer^[130]^ and cholangiocarcinoma^[131]^. In recurrent ovarian cancer, Notch3 signaling was overactive. Immunostaining for Notch3 evidenced elevated nuclear levels (up to ~4-fold) in recurrent ovarian serous carcinoma tissues compared to primary carcinoma tissues from the same patients. Furthermore, aberrant activation of Notch3 was associated with poor prognosis^[130]^. Overexpression of NCID3 in a normal ovarian epithelial cell line (IOSE-80pc) or a low-grade serous ovarian carcinoma cell line (MPSC1) increased stemness, as characterized through mRNA expression of stem cell markers as well as *ABCB1* mRNA expression, which correlated with increased resistance to carboplatin^[130]^. Expansion of a stem cell-like population of cells as well as a ABCB1^+^ cell population was also reported in pancreatic cancer, in which Notch was activated by the adipocyte hormone leptin, though co-staining for stem cell markers and ABCB1 was not performed^[132]^. In cell line models for hepatic cholangiocarcinoma, downregulation of Notch1 culminated in decreased *ABCB1* expression and sensitization to the fluoropyrimidine 5-fluorouracil^[131]^, an antimetabolite anticancer drug.

Interestingly, studies targeting upstream regulators of the Notch receptor, either through Notch ligand Jagged^[133]^ or receptor regulation^[134]^, did not observe changes in *ABCB1* or ABCB1, respectively, pointing to the involvement of the non-canonical Notch signaling pathway. Downregulation of Jagged1 in drug resistant ovarian cancer cell line SKOV3TRip2 did not alter *ABCB1* mRNA, but resulted in diminished GLI2 (Glioma-associated oncogene homolog 2), although not GLI1^[133]^. Similarly, GLI2 downregulation resulted in lowered Jagged1 levels, suggesting bidirectional regulation, and increased sensitivity to the cytotoxic drug, docetaxel. In cisplatin-resistant ovarian cancer cells (SKOV3, A2780), downregulation of caveolin-1 had no impact on total ABCB1 protein expression despite the increased apoptosis to cisplatin^[134]^, a DNA damaging agent. Caveolin-1 can indirectly upregulate Notch receptors via mitogen-associated protein kinase (MAPK) signaling and transcriptional upregulation of POFUT1, a fucosyltransferase, which in turn, activates Notch signaling, as demonstrated by increased NCID, HEY1 (Hairy Ears, Y-Linked 1), and HES1 by immunoblotting and immunofluorescence in hepatocellular carcinoma^[135]^. In addition, caveolin-1 negates γ-secretase activity, as evidenced by cleavage of amyloid-β-precursor protein and Notch^[136]^. Loss of caveolin-1 culminated in the distribution of γ-secretase to clathrin-coated non-caveolar endocytic vesicles suggesting that caveolin-1 regulates γ-secretase activity by modulating its spatial distribution, thereby impacting Notch activation.

Collectively, therefore, ABCB1 appears to be independent of ligand-Notch receptor interaction as well as Notch receptor cleavage. The transcriptional regulation of ABCB1 by Notch involves engaging non-canonical Notch signaling, which modulates other signaling networks to alter gene expression. Known non-canonical Notch targets include YY-1^[137]^, NF-κB^[138,139]^, β-catenin^[140]^, and HIF1α^[141]^, all of which are well-evidenced regulators of the *ABCB1* gene, but data are lacking for their regulation by Notch signaling in chemoresistant cancer associated with ABCB1. The lack of requirement of Notch receptor cleavage would imply the involvement of active β-catenin, which has been shown to interact with intact membrane-bound Notch^[129]^, and is strongly linked to carcinogenesis and ABCB1. Notch signaling could be engaged through active β-catenin to further strengthen the MDR phenotype and/or cell stemness.

### Hedgehog/GLI

The Hedgehog (HH) family of secreted signaling proteins were first discovered in Drosophila where it was found to function in segment polarity of larvae^[142,143]^. The vertebrate hedgehog genes, encoding three isoforms Sonic Hedgehog (SHH), Desert Hedgehog (DHH), and Indian Hedgehog (IHH), were soon identified and had similar polarizing activity^[144-146]^.

In the absence of HH, the Hedgehog pathway is maintained in an inactive state through the interaction of tumor suppressor Patched 1 (PTCH1) and the proto-oncogene Smoothened (SMO) at the plasma membrane. In ciliated cells, this occurs at the primary cilium. PTCH1 is the primary receptor for HH ligand and in the unbound state exports cholesterol from the lipid bilayer to prevent the activation of SMO, a G-protein coupled receptor belonging to the Frizzled (Fzd) family, although without direct interaction^[147]^, and keeps SMO internalized. When secreted, HH binds to two PTCH1 receptors^[148]^, disrupts cholesterol extrusion, relieves inhibition of SMO and permits changes in HH target genes^[149]^.

Central to the HH response is the nuclear translocation of glioma-associated oncogene homolog (GLI) transcription factors, which are zinc-finger proteins and whose turnover in the cytoplasm is determined by several mechanisms. Stabilization of Suppressor of fused (SUFU) through dual phosphorylation, mediated by protein kinase A (PKA) and glycogen synthase kinase3-β (GSK-3β)^[150]^, results in cleavage by the proteasome of GLI transcription factors generating repressor forms (GLI-R), which translocate to the nucleus and suppress transcriptional activation of HH/GLI target genes, such as elements of the HH signaling pathway (HH, GLI)^[151,152]^. SUFU can also sequester GLIs by direct binding^[153]^ or promote proteasome processing of the repressor form of GLI3 through the recruitment of GSK3β^[154]^. GLI transcription factors are directly phosphorylated by PKA^[155]^, MAPK^[156]^, casein kinase 1 (CK1)^[157]^ or Fused family kinases^[158]^ to affect HH signaling.

#### ABCB1 regulation by HH/GLI

Initial studies applied SHH ligand to LnCAP prostate cancer and Seg-1 oesophagus cell lines, wherein ABCB1 was upregulated and endogenous ABCB1 could be downregulated by GLI siRNA^[159]^. In chemoresistant Lucena-1 myeloid leukemia cells, PTCH1 and SMO were elevated and GLI1 was more present in the nuclei compared to their chemosensitive K562 counterparts^[160]^. Inhibition of the HH signaling pathway using cyclopamine and vitamin D3, which both bind to SMO, resulted in decreased expression of *PTCH1*, *SMO*, and *ABCB1* as well as decreased resistance to the drugs, vincristine (antimicrotubule agent), doxorubicin and mitoxantrone (anthracenedione derivative). Similar effects were observed with GLI inhibitor, Gant61, indicating regulation of ABCB1 by HH/GLI signaling, Interestingly, cyclopamine did not increase chemosensitivity in several further cancer cell lines, including ACHN renal cancer, Jurkat T-lymphocytes, and PC3 prostate cancer cells, suggesting that either these cells do not have active HH/GLI signaling or chemoresistance is governed by other transcription factors^[160]^. A comparative study for PTCH1, SMO, and nuclear GLI expression between the cell lines was not performed.

Recent studies have identified canonical and non-canonical GLI consensus sequences in multiple ABC transporters, including ABCB1, in chemoresistant cancer cells. In agreement with Queiroz *et al*.^[160]^, high GLI expression was observed in Colo205 that could be increased by treatment with 5-fluorouracil and oxaliplatin (DNA damaging agent) in a Gant61-sensitive manner^[161]^. Chromatin immunoprecipitation (ChIP) assays in Colo205 transfected with GLI shRNA or in GLI-overexpressing HCT115 cells, which have low GLI levels, evidenced active regulation of *ABCB1* and ABCB1 by GLI, as previously described for electromobility shift assay (EMSA) assays in ovarian cancer cells^[162]^. In the presence of GLI shRNA, decreased GLI binding to the *ABCB1* promoter region was observed, which was in line with decreased transcriptional activity, assessed by pull-down with anti-acetyl-H3 antibody. Analogous to GLI, ABCB1 was elevated by 5-fluorouracil and oxaliplatin, and basal levels could be attenuated by Gant61. In overexpression studies, increased GLI promoter binding was correlated with augmented transcriptional activity, and increased mRNA and protein expression of ABCB1. *In silico* analysis of patient-derived mRNA expression profiles from three different databases proved inconclusive for *ABCB1*: *ABCB1* was lower in two datasets and higher in the third . Moreover, *ABCB1* was linked to poorer prognosis lower survival in two out of the four datasets analyzed^[161]^. The significance of HH/GLI signaling for ABCB1 expression is further exemplified in the effectiveness of HH pathway inhibitors in reducing ABCB1 protein expression and/or activity^[163-165]^, as well as reducing tumors and prolonging survival in a mouse medulloblastoma model^[166]^.

The β-1,4-galactosyltransferase (B4GALT) family of enzymes is responsible for the synthesis of complex N-linked oligosaccharides present in many glycoproteins as well as for the generation of glycolipids. B4GALT has also been reported to affect the HH signaling pathway, leading to increased MDR in both drug resistant leukemia cell lines and patient samples from acute and chronic myeloid leukemias^[167,168]^. Overexpression of B4GALT1 or B4GALT5 in HL60 cells resulted in increased expression of key HH signaling components (PTCH1, SMO, GLI-1) as well as ABCB1 and ABCC1, in a cyclopamine-sensitive manner. Furthermore, B4GALT1 and B4GALT5 were elevated by more than 2-fold in > 50% of patient samples exhibiting MDR^[167]^. With their key role in glycosylation, B4GALT enzymes might either directly affect PTCH1 or SMO synthesis or activity through increased glycosylation. Alternatively, elevated glycolipids could aid in the clustering of cholesterol and impact PTCH1-SMO signaling in the membrane to favor signal transmission.

Sustained HH signaling was found to be the consequence of lowered PTCH1 levels, regulated by microRNA, in glioblastoma multiforme. Repression of PTCH1 in glioblastoma cells exhibiting resistance to temozolomide (a DNA alkylating agent) could be attributed to the microRNA (miRNA), *hsa-mir-9-(1-3)*, which targeted PTCH1 mRNA, and was confirmed in human tissue samples using *in silico* analysis^[169]^. In contrast, in multiple myeloma cells and flank mouse models, deregulated HH signaling with downstream increase in proliferation, downturned spontaneous apoptosis and increased drug resistance were a result of augmented autocrine signaling of HH ligands, which are increased in CD138^+^ multiple myeloma cells^[170]^. The authors did not observe an increase in *ABCB1*, but did not test *ABCC1* or *ABCG2*. Rather, the drug resistance acquired was explained by increased expression of the antiapoptotic protein, Bcl-2.

### Wnt/β-catenin

#### The Wnt pathways

The Wnt signaling pathway controls cell proliferation and body patterning throughout the development of both vertebrates and invertebrates. It plays a key role in body axis formation (reviewed in^[171-173]^). Several branches of the Wnt-mediated signaling cascade have been described^[41,174-176]^. In mammals, the most prominent is the canonical Wnt pathway that mediates activation of the β-catenin/TCF/lymphoid enhancer factor (TCF/LEF) transcriptional machinery^[41,177]^. There are three non-canonical pathways: the JNK/planar cell polarity (PCP) pathway, a Ca^2+^ releasing pathway for cell motility and adhesion, and a PKA-dependent pathway for myogenesis^[178]^.

#### The canonical Wnt pathway

Wnts are secreted cysteine rich glycoproteins that are essential for a wide array of developmental and physiological processes. Currently, 19 human Wnt genes are identified^[179]^. The Wnt proteins signal across the plasma membrane by interacting with Wnt receptors, which consist of a heterodimeric complex of Fzd receptors and members of the low-density-lipoprotein-related protein (LRP) family, such as LRP5/6^[180]^. This trimeric complex formation is a prerequisite for Wnt signaling.

Inhibitors of Wnt signaling belong to small protein families, including soluble Fzd related proteins (sFRP), Dickkopf (Dkk), and Wnt inhibitory factor (WIF) (reviewed in^[41,181,182]^). Their common feature is to antagonize Wnt signaling by preventing ligand-receptor interactions or Wnt receptor maturation. Conversely, the Wnt activators, R-spondin and Norrin, promote Wnt signaling by binding to Wnt receptors or releasing a Wnt-inhibitory step. Recent studies have uncovered the Lgr5 family, whose members bind R-spondins with high affinity to potently enhance Wnt signals, in adults (reviewed in^[183,184]^) as well as CSCs^[185,186]^.

In the absence of Wnts, cytoplasmic β-catenin is tagged for degradation by a multi-protein degradation (“destruction”) complex orchestrated by the tumor suppressor protein, Axin. Axin acts as a scaffold for this complex by directly interacting with its other components, adenomatous polyposis coli (APC) and the kinases, GSK3β and CK1, which constitutively phosphorylate β-catenin to promote subsequent ubiquitylation and continuous elimination by the ubiquitin-proteasome pathway (reviewed by^[187]^).

When Wnt ligands bind to the Fzd-LRP receptor complex, the cytoplasmic tail of LRP5/6 is phosphorylated by GSK3β and CK1, resulting in the binding of Axin^[188,189]^. In a process that involves activation of the cytoplasmic protein, Dishevelled (Dvl), this leads to disruption of the destruction complex, promoting stabilization, accumulation, and nuclear translocation of the co-activator β-catenin, where it triggers transcription of target genes by associating with transcription factors TCF/LEF^[190-192]^ (reviewed in^[171,193]^). In the absence of Wnt signaling, TCF/LEF repress target genes, helped by transcriptional co-repressors such as transducin-like enhancer protein (TLE)/groucho(Gro) to silence Wnt responsive genes^[194,195]^. Upon Wnt signaling, nuclear β-catenin displaces Gro from TCF/LEF and recruits transcriptional coactivators and histone modifiers, Bcl9, Pygopus and CREB-binding protein (CBP)/p300^[196-198]^, which form a multimeric complex with TCF/LEF to drive expression of genes, such as c-myc, cyclin D1, and *ABCB1*^[199-202]^. More Wnt target genes can be found on the Wnt homepage^[203]^.

#### Wnt signaling and ABCB1-dependent MDR in cancer

Since the initial landmark study on *APC* mutations in the development of colorectal carcinogenesis^[204]^ (reviewed in^[205,206]^), aberrant Wnt signaling has been shown to affect many cancer tissues (reviewed in^[176-178,207-211]^). Because Wnt signaling is a key driver of most types of tissue stem cells^[212]^, aberrant Wnt signaling plays an important role in the induction and maintenance of cancer stemness^[209]^.

As a rule of thumb, Wnt contributes to carcinogenesis after genetic mutations and epigenetic mechanisms affecting pathway components, resulting in altered expression of Wnt relevant genes, including *ABCB1*^[208,209,213]^. Both mechanisms either positively or negatively interfere with the Wnt pathway, depending on whether stimulatory or inhibitory regulators of Wnt signaling are targeted. Cooperativity between the Wnt signaling and various other developmental signaling pathways has also been implicated in promoting or even potentiating carcinogenesis, such as NF-κB (see^[208]^ and below).

Various epigenetic control mechanisms affect Wnt signaling to alter *ABCB1* target gene expression and MDR [Table 1]. CpG island hypermethylation is important for gene inactivation in cancer cells and has been described in almost every type of tumor (reviewed in^[214,215]^). It affects various gene loci of the Wnt/β-catenin pathway, thereby modulating *ABCB1* expression and includes *APC* (see^[216-219]^), upstream modulators such as cyclooxygenase 2 (*PTGS2*)^[216,219]^ or *SFRP5*^[220]^, and/or target genes including the *ABCB1* gene locus^[216-219]^ in various malignancies, e.g, prostate adenocarcinoma, non-small cell lung cancer, or leukemia cells.

**Table 1:** Regulation of Wnt signaling and target gene *ABCB1* by ncRNAs in malignant tissues and cells

ID*	Expression in malignant tissue/cell	Malignant tissue/cell	Effect on ABCB1 expression	Target gene	Effect on target gene expression	Mechanism responsible for ↑ Wnt signaling (Wnt) and ABCB1 (direct/indirect)	Ref.
miRNAs							
hsa-mir-451a	↓	Colorectal CSC	↑	*MIF* (Macrophage migration inhibitory factor)	↓ (mRNA + protein)	MIF↑→COX2↑→Wnt↑→ABCB1↑	[336]
hsa-mir-27a	↓	Hepatocellular carcinoma	↑	*FZD7* (Frizzled 7)	↓ (protein)	Fzd7↑→Wnt↑→ABCB1↑	[337]
hsa-mir-33a	↓	Pancreatic ductal adenocarcinoma	↑	*CTNNB1* (β-catenin)	↓ (mRNA + protein)	β-catenin ↑→Wnt↑→ABCB1↑	[338]
hsa-mir-134	↓	Oral squamous CSC	↑	n.d.	n.d.	Wnt↑→ABCB1↑	[339]
hsa-mir-506	↓	Colorectal carcinoma	↑	n.d.	n.d.	Wnt↑→ABCB1↑	[340]
hsa-mir-122	↓	Hepatocellular carcinoma	↑	n.d.	n.d.	Wnt↑→ABCB1↑	[341]
lncRNAs							
CCAL	↑	Colorectal carcinoma	↑	*TFP2A* (Activating Enhancer Binding Protein 2 Alpha)	↓ (protein)	TFP2A↓→Wnt↑→ABCB1↑	[231]
PVT1/HSA-LNCG007059	↑	Bladder urothelial carcinoma	↑	n.d.	n.d.	Wnt↑→ABCB1↑	[342]
HOTAIR/HSA-LNCG003959	↑	Non-small cell lung carcinoma	↑	n.d.	n.d.	Wnt↑→ABCB1↑	[343]
GAS5/HSA-LNCG004395	↓	Breast cancer	↑	hsa-mir-221-3p	↓ (miRNA)	hsa-mir-221-3p↓→DKK2↓→ Wnt↑→ABCB1↑	[232]
CRNDE/HSA-LNCG006310	↑	AML	↑	n.d.	n.d.	Wnt↑→ABCB1↑	[344]

*miRbase database; **lncRNome database; AML: Acute myeloid leukemia; CSC; cancer stem cell; n.d.: not determined.

Acetylation and deacetylation are counteracting, post-translational epigenetic modifications that affect various histone and non-histone proteins^[221]^, whereby acetylation by histone acetyl transferases (HATs) increases transcriptional activation and deacetylation by histone deacetylases (HDACs) is associated with transcriptional deactivation^[222]^. In a study performed on breast cancer cells, binding of hyaluronan to the CSC marker, *CD44*^+^, a target gene of Wnt signaling^[223]^, upregulated HAT CBP/p300, thus promoting acetylation of β-catenin and the inflammatory transcription factor, NF-κB-p65, leading to activation of TCF/LEF and NF-κB-specific transcription. This resulted in upregulation of *ABCB1* and the anti-apoptotic gene, Bcl-xL (*BCL2L1*), and promoted chemoresistance in MCF-7 cells^[224]^. Conversely, activation of the HDAC Sirtuin 1, by the antioxidant resveratrol, prevented these effects with consequent chemosensitivity and caspase-3 mediated apoptosis.

Among non-coding RNAs (ncRNAs)^[225,226]^, miRNAs are ~22 nucleotide RNAs that function by direct RNA silencing and post-transcriptional regulation of mRNA targets^[227]^. miRNAs function via complementary base-pairing with sequences within mRNA molecules, which results in repression of protein synthesis. When they are complexed with Argonaute protein, miRNAs use seed sequences near their 5’ end to base pair with a target mRNA to induce deadenylation and decay or translational regulation^[227,228]^. In various MDR cancers, lower levels of miRNAs targeting mRNAs of Wnt/β-catenin signaling components were associated with increased *ABCB1* expression [Table 1].

Long non-coding RNAs (lncRNAs) (> 200 nucleotides) are autonomously transcribed RNAs found in the nucleus, cytoplasm, or both, that do not encode proteins and their specific functions are still under investigation^[229,230]^. Nevertheless, several studies have investigated the role of various lncRNAs on Wnt/β-catenin signaling and ABCB1 expression in cancer [Table 1]. In most of these reports, increased levels of lncRNAs resulted in activation of the Wnt pathway, increased *ABCB1* expression, and MDR. In some instances, the complexity of lncRNA functions became apparent by indirect effects on Wnt signaling, e.g, via interactions with epigenetic or miRNA regulation. Upregulation of the lncRNA *CCAL* induced by decreased CpG island methylation and increased acetylation of the *CCAL* promoter region enhanced the development of colorectal cancer by increased proteasomal degradation of transcription factor AP-2a, which derepresses Wnt/β-catenin signaling and *ABCB1* expression^[231]^. In adriamycin resistant MCF-7 cells and breast cancer tissues, decreased levels of the lncRNA *GAS5* suppressed the expression of miRNA *hsa-mir-221-3p*, which relieves expression of the Wnt inhibitor DKK2, resulting in increased Wnt signaling and ABCB1 upregulation^[232]^.

Genetic alterations of the Wnt pathway components are found in most cancer studies on ABCB1 expression and MDR, which are under the control of Wnt signaling. They comprise mutations, deletions or amplifications, resulting in enhancement or reduction/loss of activity of ligands, receptors, and its cytosolic or nuclear components [Table 2] (reviewed in^[176,208,209]^).

**Table 2:** Genetic and expression changes of Wnt signaling in malignant tissues and cells and their impact on target gene ABCB1

Affected Wnt signaling gene	Expression/Function in malignant tissue/cell	Malignant tissue/cell	Effect on ABCB1 expression	Mechanism responsible for ↑ Wnt signaling (Wnt) and ABCB1 (direct or indirect)	Ref.
*WNT3a*	↑	Glioblastoma	↑	Wnt↑→ABCB1↑	[114]
*WNT5a*	↑	Uterus sarcoma & breast cancer	↑	Hypomethylation WNT5A→PKA↑→CRE/TCF↑→ ABCB1↑	[233]
*FZD1*	↑	Neuroblastoma AML Breast cancer	↑	Wnt↑→ABCB1↑	[345-347]
*FZD7*	↑	Esophageal squamous cell Hepatocellular	↑	Wnt↑→ABCB1↑	[348,349]
*LGR5*	↑	Colorectal CSC	↑	Wnt↑→ABCB1↑	[105]
*DVL1-3*	↑	Colorectal	↑	Nuclear Tcf4/β-catenin complex↑→ABCB1↑	[350]
*APC*	↓*	Colorectal CSC	↑	Wnt↑→ABCB1↑	[351]
*CTNBB1*	↑ ↑** ↑	Colorectal CSC Colorectal CML	↑	Wnt ↑→ABCB1↑ Wnt ↑→ABCB1↑ Nuclear Tcf4/β-catenin complex↑→ABCB1↑	[352-354]
*CREBBP*	±	Uterus sarcoma Erythroleukemia Colorectal adenocarcinoma	↑	MEK_1/2_/ERK_1/2_↑→Nuclear Tcf4/β-catenin/CBP complex↑→ABCB1↑	[355]
*PYGO2*	↑	Breast cancer Brain glioma	↑	Nuclear Tcf4/β-catenin/PYGO2 complex↑→ABCB1↑	[356,357]

*nonsense and frameshift mutations; **in-frame deletion; AML: Acute myeloid leukemia; CML: chronic myeloid leukemia; CSC: cancer stem cell; n.d.: not determined.

Examples of other Wnt dependent mechanisms of increased ABCB1 expression are described as follows. Wnt5a is upregulated in MDR uterus sarcoma and breast cancer cells, and is associated with hypomethylation of CpG islands of a *Wnt5a* intron sequence^[233]^. Wnt5a increases cAMP response elements and TCF/LEF transcriptional activity, ABCB1 and chemoresistance in MDR cancer cells, suggesting that PKA dependent non-canonical Wnt signaling also regulates ABCB1 expression^[233]^. Chronic inflammation and oxidative stress are common and co-substantial pathological processes accompanying and contributing to cancers^[234]^. The pro-inflammatory transcription factor, NF-κB-p65, upregulates *PTGS2*^[235]^ and *ABCB1*^[139,236]^. Coincidently, both *PTGS2* and *ABCB1* are also target genes of Wnt/β-catenin ^[202,237,238]^. Gutkind *et al.*^[239]^ first demonstrated that prostaglandin E2 (PGE2), the product of cyclooxygenase 2, enhances colon cancer progression by binding with the G protein-coupled receptor, EP2, by a signaling route that involves the activation of phosphoinositide-3-kinase (PI3K) and AKT/PKB, leading to the inactivation of Axin and release of GSK3β from its complex with Axin. This process relieves the inhibitory phosphorylation of β-catenin and consequently upregulates *PTGS2* and *ABCB1*^[239]^. Although initially described for colorectal cancer progression, this mechanism has been described in other cancer types^[240-243]^. This positive feedback loop (via upregulation of *PTGS2*) would further enhance aberrant Wnt signaling in affected cancer tissues, and possibly ABCB1 upregulation. Interestingly, this cooperative inflammatory and carcinogenic signaling mechanism has led to promising therapeutic anti-cancer concepts with nonsteroidal anti-inflammatory drugs (reviewed in^[244-246]^).

### Other developmental signaling pathways

Additional transcription factors or aberrant epigenetic misregulation have been reported to regulate *ABCB1* expression and/or activity.

#### Bone morphogenetic proteins & transforming growth factor β

The TGFβ superfamily comprises multiple ligands, such as BMPs, TGFβ, Activins as well as Nodal and Lefty, and their corresponding receptors at the cell surface^[247]^. In embryonic development, they are required for axis formation, left-right-patterning, organ asymmetry, gastrulation, and organogenesis (reviewed in^[248]^). Upon ligand binding, a heteromultimeric receptor complex transduces signals to the intracellular milieu via receptor-activated Smad proteins, which are phosphorylated through the acquired serine/threonine kinase receptor activity^[249,250]^. TGFβ, Nodal, and Activin receptors generally activate Smad2/3, whereas BMP receptors, which do not exhibit cooperative assembly, use Smad1/5/8, though other combinations of receptors and Smads have also been observed^[251,252]^. Cytosolic co-Smad4 is recruited to the phosphorylated Smads forming a complex that is exported out of the nucleus at a slower rate than monomeric Smads, leading to their accumulation in the nucleus^[253]^ and subsequently to the elevation of transcriptionally regulating genes associated with cell proliferation, cell cycle, apoptosis, and cell differentiation. Signaling to the nucleus can also occur through Smad-independent pathways, such as MAPK and PI3K/AKT.

Deregulation of TGFβ signaling is well documented in cancer playing a role, not only in tumor cell growth and survival but also in determining tumor heterogeneity and self-renewal of CSCs (reviewed in^[254]^). Exogenous application of TGFβ induces spontaneous neoplastic transformation of hepatocytes, correlating with augmented *ABCB1*^[255]^, as well as increasing the side population, *ABCB1* mRNA, TGFβ receptor mRNA, and MAPK signaling in lymphoma cell lines^[256]^. In A549 lung cancer cells, antisense oligonucleotides targeting *hsa-mir-10a* reduced phosphorylated Smad2, survival proteins (Bcl-2, Survivin), and ABC drug transporters (ABCB1, ABCC1) with increased sensitivity to cisplatin^[257]^.

#### Fibroblast growth factor

The fibroblast growth factors (FGF) are a family of 23 known secreted growth factors recognized by five FGF receptor (FGFR) tyrosine kinases^[258]^. Ligand binding induces FGFR dimerization and can initiate several phosphorylation cascades, such as PKC, STAT (Signal Transducer And Activator Of Transcription), MAPK, and PI3K/AKT. Transcriptional regulation of genes associated with proliferation, differentiation, and growth depends on STAT1/3/5, FOXO1 (Forkhead Box O1) or ETS (V-Ets Avian Erythroblastosis Virus E26 Oncogene Homolog) transcription factor activity. In cancer, FGF signaling contributes to cell growth and survival, chemoresistance, and neoangiogenesis^[259]^.

In paclitaxel-resistant prostate cancer PC3 cells, ETS1 silencing inhibited the activity of the *ABCB1* promoter, which contains ETS binding sites, reduced ABCB1 protein, and reversed resistance to paclitaxel^[260]^, an antimicrotubule drug. ETS mediated regulation of *ABCB1* was verified in a gastric cancer cell line exhibiting enhanced *ABCB1* expression and vincristine resistance using ETS2 overexpression combined with ChIP assays^[261]^. At the level of FGF, immunohistochemical studies of bladder cancer tissue from patients showed correlation between basic FGF and ABCB1 signals^[262]^.

#### Hippo/YAP

Growth factors, signaled through cell surface receptors, and cell density, signaled through cell-cell contacts, are the major regulators of the Hippo/YAP pathway, which have multiple roles in the course of development, including growth control and morphogenesis^[263]^. When a growth signal is triggered, the transcription factor Yes-associated protein (YAP) escapes cytosolic degradation and translocates to the nucleus to drive gene transcription. When growth should be inhibited, YAP is phosphorylated^[264,265]^, retained in the cytosol by binding to 14-3-3, or degraded by the proteasome. Though there are indications in the literature that ABCB1 mRNA and protein are driven by Hippo/YAP signaling in both tumor cells^[266,267]^ and ovarian CSCs^[268]^, further experimental evidence is required to ascertain direct or indirect as well as positive or negative transcriptional regulation.

#### YBX1

Y-box binding protein 1 (YBX1 or YB-1) is a transcription factor, expressed at various stages of development and in early hematopoiesis^[269,270]^, but is also active in tumor cells^[271-273]^. Deregulation of YBX-1 in carcinogenesis seems to be attributed to a combination of epigenetic alterations and MAPK signaling^[274-276]^. Furthermore, YBX1 binds to the promoter region of ABC transporters, including ABCB1, and predicts poor outcome (reviewed in^[277]^). Moreover, YBX1 transcriptionally regulates genes involved in cell proliferation^[271]^, cell cycle^[278]^ and metabolism^[273]^, which can, in turn, impact ABCB1 expression and activity, e.g., GSK3β^[271]^ or NF-κB^[279]^.

#### Snail

The Snail transcription factors (SNAI1-3) are well evidenced in epithelial-mesenchymal transition (EMT) whereby metastatic tumor cells acquire the ability to detach from the tumor mass^[280,281]^. In embryonic development, Snail is intimately involved in early patterning^[282]^, most likely through transcriptional repression^[283]^ and conferring resistance to cell death by cell cycle inhibition^[284]^. Epigenetic alterations, such as changes in non-coding mRNA or DNA methylation status, are the major contributors to the deregulation of Snail in cancer (reviewed in^[285]^).

In hepatocellular carcinoma cell lines (MHCCLM3, SMMC-7721), MDR was increased by augmented levels of *ABCB1*, ABCB1 and *ABCG2* when SLUG (*SNAI2*) was downregulated. *ABCB1* promoter luciferase assay and promoter sequence analysis predicted *SNAI2* promoter binding^[286]^. Though these observations do not align with the EMT role of Snail family members, the authors suggest a tumor suppressor role for SNAI2 that is tumor-type specific^[286]^. Conversely, in colorectal cancer, elevated Snail correlated positively with tumor size and metastatic nature in patient tissue samples. Snail overexpression in HCT116 and SW480 cells resulted in upregulation of ABCB1 mRNA and protein without significant effects on other relevant MDR ABC transporters (ABCCs, ABCG2). *ABCB1* promoter luciferase and ChIP assays evidenced direct binding of Snail to the *ABCB1* promoter^[287]^, indicating that Snail and ABCB1 promote EMT. The transcription repressor function of Snail does not align with the positive transcription regulation of *ABCB1*. However, rather than simply preventing transcription through blockade of the RNA polymerase, repressors can also modify DNA looping by binding at multiple DNA sites, priming a transcription site to modulate cellular responsiveness or fine-tuning the transcriptional response^[288]^.

## A novel developmental transcriptional regulator of ABCB1 in cancer: PITX2

The paired-like homeodomain (PITX) subfamily of bicoid class homeodomain proteins consists of three paralogues, PITX1, PITX2, PITX3, which have essential roles in embryonic development, including organ asymmetry^[289]^, through their function as transcriptional regulators. The PITX proteins harbor a highly homologous homeobox domain (97% homology), in which a crucial lysine residue is expressed at residue 50, which is determinant of DNA binding specificity^[289]^. A high degree of similarity is also seen in the C-terminus (55%-70%) which is required for protein-protein interactions, such as dimerization with other PITX proteins. The N-terminus shows fewer common amino acids with homology varying from 18%-31%^[289]^. PITX2 undergoes alternative splicing and the use of different promoter regions results in four isoforms (PITX2A, PITX2B, PITX2C, PITX2D)^[290,291]^, which exhibit isoform transcriptional specificity, sometimes working in synergism, for target genes in embryonic development and cellular functions^[292]^. For example, PITX2B regulates heart asymmetry^[293]^, while PITX2A alters cytoskeleton and migration properties^[294]^, and PITX2C preferentially activates atrial natriuretic factor expression in cardiogenesis^[295]^. PITX2D does not harbor transcriptional activity since the homeodomain is non-functional. Rather, PITX2D suppresses transcriptional activity of other PITX2 isoforms through interaction and formation of heterodimeric complexes^[292]^. PITX2 isoforms can also work synergistically depending on the targeted promoter^[292]^.

PITX1 governs the development of anterior structures and the brain, including the pituitary gland; PITX2 determines organ asymmetry and pituitary gland development whereas PITX3 is involved in lens formation and maintenance of midbrain dopaminergic neurons^[289,296]^. *Pitx2* knockout mice are embryonically lethal due to developmental deficits in multiple organs^[297]^ whereas *Pitx1* knockout mice die after birth^[298]^. In adults, PITX2 activity is restricted to selective tissues, appearing to be important in cardiac injury recovery^[299]^ and maintenance of mature pituitary function^[300]^. The increase in cell death occurrence during development in *Pitx2* knockout mice serves as an indication of its potentially pivotal role in governing cell life and death^[300]^. Mutations in *PITX2* manifest in autosomal dominant Axenfeld-Rieger syndrome, a group of diseases affecting the development of the anterior segment of the eye^[301,302]^.

### PITX2 in cancer

Though PITX1 has been linked to cancer, it has anti-tumorigenic properties^[303-305]^ and a role for PITX3 has not been well-established. In contrast, *PITX2* was identified as a target gene of human acute leukemia ALL1 in *ALL1*^-/-^ embryonic stem cells. Furthermore, its downregulation in leukemia cell lines with ALL1-inactivating chromosomal rearrangements was indicative of the oncogenic property of PITX2/ARP1 in leukemia^[306]^, possibly in conjunction with hypermethylation of its promoter, observed in acute myeloid leukemia^[307]^. Early studies linked PITX2 to Wnt/β-catenin signaling, which is frequently deregulated in tumorigenesis, either by acting as a β-catenin target gene^[308]^ or working synergistically with β-catenin to regulate promoter activity and hence gene expression^[309]^. Subsequently, PITX2 mediated regulation of cyclin A1^[310]^, cyclin D2/*CCND2*^[308,311]^, cyclin D1/*CCND1*, and c-Myc/*MYC*^[312]^ has been observed. Though increased PITX2 has been associated with cancer progression in various tumors^[313-315]^, the subset of genes regulated by PITX2 appears to be cancer specific. The presence of PITX2 in sites outside of the primary tumor suggests its role in invasion and metastatic potential^[316]^.

Several cellular factors could promote aberrant PITX2 expression in cancer. *In silico* analysis found the highest *PITX2* RNA changes in human colon adenocarcinomas^[43]^ and could be attributed to APC, a β-catenin suppressor frequently mutated in colon cancer, thus driving β-catenin excess, nuclear translocation, and transcriptional upregulation of *PITX2*^[308]^. As mentioned above, DNA methylation patterns are tumor-specific and impact cellular architecture. Hypermethylation of the *PITX2* promoter, resulting in decreased PITX2 expression, has predicted survival^[317,318]^, but has also been associated with poor prognosis^[307,319]^, and is a prospective prognostic biomarker for prostate cancer^[320]^. Recent evidence points to miRNA-mediated regulation of PITX2. *PITX2* is negatively regulated by miRNAs^[321]^, but PITX2 can also regulate miRNAs to effectuate its downstream effects, such as augmented cell proliferation^[322]^. Potential additional malfunctioning mechanisms involved in aberrant oncogenic PITX2 include mRNA export, translation initiation factors, protein folding, and proteolysis in the cytosol and nucleus.

Upregulation of PITX2 appears to be an early event in tumorigenesis observed in tumor tissue sample at early stages^[43]^, suggesting that PITX2 could increase susceptibility or permissibility to manifestation of mutation events or adaptive mechanisms by upregulating genes favoring tumor progression such as cell cycle control genes^[312,314,322]^, proto-oncogenes^[312,314]^, EMT markers^[315]^, and MDR mediating factors^[42]^. Moreover, PITX2 could transcriptionally repress tumor counteracting/suppressor genes or epigenetically alter gene expression^[323,324]^. Regulation of PITX2 cytoplasmic-nuclear shuttling remains uninvestigated. Evidence for its multifunctional C-terminal tail, which has protein interaction capabilities^[325]^, a potential nuclear localization sequence^[326]^, and PKC phosphorylation sites^[327]^ suggests that PITX2 could be regulated analogously to other related HOX transcription factors, namely through cytosolic levels.

### PITX2 regulation of ABCB1

In renal cell carcinoma (RCC) and colon cancer cell lines exhibiting MDR and augmented ABCB1 expression, we have identified PITX2 as a contributor to chemotherapeutic drug resistance through transcriptional upregulation of *ABCB1*^[42]^ as well as *ABCC1*, *ABCG2*, and the drug uptake transporter, *SLC22A3*^[43]^. The PITX2 consensus sequence, *TAATCC*, was found at -7,626 bp and -14,510 bp in the *ABCB1* promoter region^[42]^. Using ChIP assays by pulling down overexpressed myc-tagged PITX2C^[42]^ or endogenous PITX2^[43]^, PITX2 was evidenced to bind to both sites in chemoresistant colon and renal cancer cell lines and was supported by RNAi studies wherein *PITX2* downregulation resulted in attenuated *ABCB1* mRNA and ABCB1 protein as well as chemoresistance^[42,43]^. The action of PITX2 on ABCB1 is independent of β-catenin since *PITX2* overexpression in β-catenin-deficient mouse keratinocytes increased *ABCB1* expression, cell survival, and chemoresistance^[42]^ and the GSK3β inhibitor, SB216763 (which would augment β-catenin stabilization), decreased *PITX2* promoter activity^[43]^.

Though all PITX2 isoforms can upregulate ABCB1, synergism of PITX2 isoforms on drug transporters was not evident; PITX2C had the greatest effect on ABCB1 upregulation and resistance to vincristine in RCC^[43]^. The effectivity of PITX2C was confirmed in Caki-1 cells, derived from a metastatic skin site of a primary renal tumor, where PITX2C maximally increased promoter activity of cyclin D1/*CCND1* and mRNA expression of select PITX2 target genes (Aquisap, A., Zarbock, R. Lee, W.K., *unpublished data*). A recent report detailing a new role for PITX2C in gastrulation during embryonic development could underpin PITX2C’s apparent enhanced oncogenicity and/or metastatic potential. During gastrulation, cells migrate into the three primary germ layers (mesoderm, ectoderm, endoderm), a process that is driven by *pitx2c*-mediated expression of the chemokine *cxc112b* in zebrafish^[328]^. These findings reiterate the concept that specific gene subsets are regulated by each PITX2 isoform^[292]^. This is further exemplified in a study of ectopic *PITX2A*, *PITX2B*, or *PITX2C* overexpression in ovarian cancer cell lines, wherein TGFβ signaling promoted invasion and EMT was activated, but with *PITX2* isoform gene activation selectivity^[315]^. Moreover, even when all PITX2 isoforms increased prostate cancer cell mobility, only PITX2A conferred a specific mobility advantage in the presence of Wnt5a stimulation^[329]^.

The molecular identification of the reactivated PITX2 transcriptional complex in cancer is currently unknown; however, it appears that PITX2 requires additional co-factors or interaction partners to transcriptionally regulate its target genes^[330-332]^, as commonly seen in developmental PITX2 signaling with, for example, SOX2 (sex determining region Y-box 2) and LEF1^[333]^ or FOXC1 (Forkhead box C1)^[334]^. As mentioned above, the Wnt/β-catenin pathway is not a pre-requisite for PITX2, however, it could be envisaged that co-activation of Wnt/β-catenin is likely to enhance and strengthen PITX2-mediated transcription of *ABCB1* in a self-propagating cycle.

From PITX2’s role in embryonic development and reactivation in cancer, it is reasonable to assume its influence on CSCs, which, as detailed above, harbor strong defense mechanisms against chemotherapeutics. Only a single study has reported increased mutations of PITX2 in CSCs derived from patient bladder cancer tissue samples^[335]^. Using cell surface markers and flow cytometry, single cell bladder CSCs were distinguished from cancerous non-stem cells and bladder epithelial cells. Single cell sequencing identified somatic nonsynonymous mutations in PITX2 in bladder cancer tissue obtained through transurethral resection. These mutations were found exclusively in bladder CSCs, although their role in bladder CSC self-renewal and tumor propagation was not further investigated^[335]^.

## Conclusions

**Figure 1:**
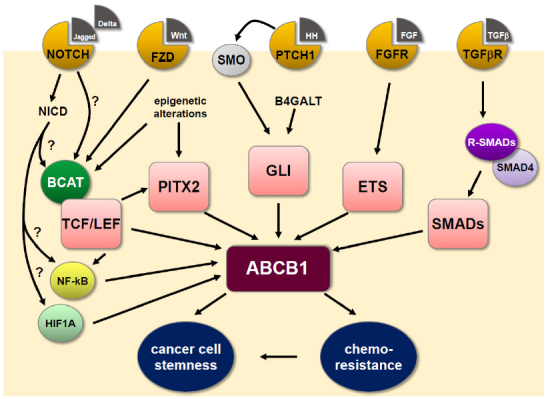
Summary of ABCB1 regulation by developmental signaling pathways in cancer. The MDR P-glycoprotein ABCB1 is central to conferring oncogenic chemoresistance and is also a phenotypical marker for cancer cell stemness. Alterations in the transcription of ABCB1 represent the major regulatory pathway and the ABCB1 promoter region harbors consensus binding sequences for numerous transcription factors, including those primarily involved in embryonic development, which can resurge during carcinogenesis. See main text for further details. B4GALT: β-1,4-galactosyltransferase; BCAT: β-catenin; ETS: erythroblast transformation specific; FGF: fibroblast growth factor; FGFR: fibroblast growth factor receptor; Fzd: frizzled; GLI: glioma-associated oncogenes; HH: hedgehog; HIF1A: hypoxia-inducible factor 1α; NF-κB: nuclear factor kappa B; PTCH1: patched 1; PITX2: paired-like homeodomain transcription factor 2; R-SMADs: receptor-activated SMADs; SMO: smoothened; TCF/LEF: T-cell factor/lymphoid enhancer factor; TGFβ: transforming growth factor β; TGFβR: transforming growth factor β receptor; Wnt: wingless-INT.

Chemoresistance develops in multiple cell populations within the heterogenic tumor. Malignant cells may display inherent or acquired MDR, depending on the tumor tissue origin and use of chemotherapeutic drugs, respectively. However, CSCs or persister cells use enhanced chemoresistance as part of their defense mechanisms to counteract cytotoxic cues, permitting their survival and regeneration of tumor tissue. Mounting evidence points to the regulation of the MDR transporter, ABCB1, by several reactivated developmental signaling pathways, as delineated in this work [Figure 1]. There are still many unanswered questions. For example, do these pathways interact in a feed-forward loop? Is the timing of pathway activation crucial to ABCB1 regulation analogous to pathway co-ordination in embryonic development? Do these pathways regulate directed cell movement within the tumor and does ABCB1 contribute to this potential mechanism? With advancing technology and model systems, it is conceivable that our improved molecular understanding of MDR phenotype regulation will help increase the effectiveness of cancer therapy.

## Declarations

### Copyright

© The Author(s) 2021.
